# The American Association of Obstetricians and Gynecologists

**Published:** 1889-10

**Authors:** 


BUFFALO MEDICAL AND SURGICAL JOURNAL
A MONTHLY REVIEW OF MEDICINE AND SURGERY.

*EDITORS:*
THOS. LOTHROP, M. D. - - - W. W. POTTER, M. D.

All communications, whether of a literary or business character, should be addressed to the editors: 284 FRANKLIN STREET, BUFFALO, N. Y.

### Editorial.

### THE AMERICAN ASSOCIATION OF OBSTETRICIANS AND GYNECOLOGISTS.

THIS Association, that was organized in this city April 19, 1888, has lately held its second annual meeting in Cincinnati, O. Some features connected with this Association and its annual meeting deserve something more than ordinary notice at the hands of the medical press, and merit especial attention of the medical profession; hence we venture to point them out at this time.

When this Association was organized, it is a well-known fact that the branch of medicine which is its special province to cultivate, had been denied representation in the Congress of American Physicians and Surgeons, by reason of the action of the American Gynecological Society, which had refused to enter that body. That society not only declined to take part in its proceedings, but it appointed its annual meeting to be held in Boston simultaneously with the meeting of the Congress in Washington, thereby clearly indicating that it would "have none of it."

As soon, however, as it became known that a movement was on foot to organize another society, it canceled its former action in appointing its meeting in Boston, and agreed, by letter vote, to assemble in Washington simultaneously with the Congress. It held its meeting as appointed, ate its political crow, swallowed the Congress, without a pang of indigestion. Meanwhile the new organization was perfected, held its first annual meeting in Washington at the time of the Congress, and its first volume of transactions has gone out to receive the verdict of the profession.

The following, from the New York *Medical Journal* for August 31, 1889, in a notice of the Transactions for 1888, is introduced here as an index of what that verdict is:

This new Society has every reason for satisfaction as to both the matter and the manner in the first volume of its transactions. With the array of distinguished names in the list of fellows, and the good work with which its career has been inaugurated, the Society has reason to expect a useful and creditable future. We might select a number of papers or discussions from the volume, and label them classical. The amplest justification for the existence of any society is furnished by work such as this.

We have failed to note, thus far, a single adverse opinion or criticism thereupon.

Meanwhile the second annual meeting of this body has lately been held, and its work will soon be accessible to the medical profession throughout the world. We bespeak for it a patient and thoughtful examination, as well as an impartial criticism.

